# Using large language models to extract plant functional traits from unstructured text

**DOI:** 10.1002/aps3.70011

**Published:** 2025-06-03

**Authors:** Viktor Domazetoski, Holger Kreft, Helena Bestova, Philipp Wieder, Radoslav Koynov, Alireza Zarei, Patrick Weigelt

**Affiliations:** ^1^ Department of Biodiversity, Macroecology, and Biogeography University of Göttingen Göttingen Germany; ^2^ Campus‐Institute Data Science Göttingen Germany; ^3^ Centre of Biodiversity and Sustainable Land Use University of Göttingen Göttingen Germany; ^4^ Gesellschaft für wissenschaftliche Datenverarbeitung mbH Göttingen Göttingen Germany; ^5^ Department of Environmental Science, Radboud Institute for Biological and Environmental Sciences Radboud University Heyendaalseweg 135 Nijmegen 6525AJ The Netherlands

**Keywords:** automatic trait extraction, biodiversity, functional plant ecology, large language models, natural language processing, vascular plants

## Abstract

**Premise:**

Functional plant ecology seeks to understand how functional traits govern species distributions, community assembly, and ecosystem functions. While global trait datasets have advanced the field, substantial gaps remain, and extracting trait information from text in books, research articles, and online sources via machine learning offers a valuable complement to costly field campaigns.

**Methods:**

We propose a natural language processing pipeline that extracts traits from unstructured species descriptions by using classification models for categorical traits and question‐answering models for numerical traits. The pipeline's performance is evaluated on two large databases with over 50,000 species descriptions, utilizing approaches ranging from a keyword search to large language models.

**Results:**

Our final optimized pipeline used a transformer architecture and obtained a mean precision of 90.8% (range 81.6–97%) and a mean recall of 88.6% (77.4–97%) across five categorical traits, representing a 9.83% increase in precision and 42.35% increase in recall over a regular expression‐based approach. The question‐answering model yielded a normalized mean absolute error of 10.3% averaged across three numerical traits.

**Discussion:**

The natural language processing pipeline we propose has the potential to facilitate the digitization and extraction of large amounts of plant functional trait information residing in scattered textual descriptions.

To address fundamental questions about the role of plants in ecosystem functioning, it is necessary to understand plant species functionally (Antonelli et al., 2023). However, for many of the more than 380,000 vascular plant species already described, the ability to draw solid inferences on the spatial distribution of traits, their responses to the environment, and their importance for ecological processes is hampered by biases in the available data (Maitner et al., 2023), as well as a lack of data on characteristics that affect organismal performance, survival, development, growth, and reproduction (i.e., functional traits; Violle et al., 2007). Several large databases contain information on plant traits, including the TRY initiative (Kattge et al., 2011, 2020), the Global Inventory of Floras and Traits (GIFT) (Weigelt et al., 2020), the Botanical Information and Ecology Network database (BIEN) (Maitner et al., 2018; Gallagher et al., 2020), and the AusTraits database (Falster et al., 2021). These databases hold information on hundreds of thousands of plant species and thousands of traits—varying from individual measurements to species‐level information, and from physiological, chemical, and genomic traits to whole‐plant and structural traits—making them valuable resources for ecology (König et al., 2019). These data have facilitated a vast number of studies in functional ecology and enhanced our understanding of the global distribution of plant form and function (Wright et al., 2004; Moles et al., 2007; Díaz et al., 2016).

Although the available trait databases contain a large amount of data, only a small fraction of all plant species are covered (global mean trait completeness of 3.48% across 53 traits with data for at least 1% of the species in the TRY database; Kattge et al., 2020; Maitner et al., 2023). Furthermore, the information contained in these databases is strongly biased on multiple levels (Meyer et al., 2016; König et al., 2019; Maitner et al., 2023). For instance, while information for traits such as growth form may be well represented, other, equally important traits such as specific leaf area may not. In addition, the availability of traits is taxonomically and spatially biased due to influence from socioeconomic drivers (Maitner et al., 2023). While trait imputation based on trait–trait correlations and phylogenetic relationships among species may help to fill gaps (Schrodt et al., 2015), it only works for taxonomically and geographically representative data (Penone et al., 2014). Thus, the mobilization of more trait data holds great potential, particularly for analyses at a fine spatial grain (Bruelheide et al., 2018), high trait resolution (i.e., many specific traits), or in underrepresented regions (e.g., the tropics).

New trait data may come from extensive field campaigns and ecological experiments; however, these approaches, while essential, are time‐consuming and costly. A promising complementary approach for certain species and traits that is potentially faster and cheaper is to mobilize already sampled but so far untapped information hidden in the wealth of published and online literature. Regional floras, checklists, and taxonomic monographs, for example, contain information on morphological, reproductive, dispersal, and other traits (Weigelt et al., 2020). Additionally, a huge amount of information is contained in primary research articles (e.g., species descriptions) and many regional to global websites (e.g., Wikipedia, Wikimedia Commons, JSTOR Plant Science, Plants of the World Online). Until recently, such data had to be manually extracted page by page to locate specific trait information, but the emergence of powerful machine learning (ML) techniques has opened new avenues for the automated extraction of this information at a large scale (Folk et al., 2024).

In the past decade, there has been an exponential rise in the application of ML for a variety of tasks, including ecological questions (Thessen, 2016). As data sources continue to grow, the ability to automatically learn patterns from data becomes increasingly critical. Deep learning (DL) models utilize large numbers of hidden layers and nonlinear activations (LeCun et al., 2015) and have sped up progress in computer vision, digital signal processing, and natural language processing (NLP). Ecologists have only recently started using ML and DL (Christin et al., 2019; Pichler and Hartig, 2023), particularly for tasks in wildlife conservation (Tuia et al., 2022) like animal detection in camera traps (Steenweg et al., 2017) and species and individual recognition in bioacoustic signals (Sugai et al., 2019). In functional ecology, convolutional neural networks (CNN) have been used, for example, to predict trait values by coupling images from iNaturalist and traits from the TRY database (Schiller et al., 2021) and to measure functional traits of skeletal museum specimens (Weeks et al., 2023).

The field of NLP operates at the intersection of diverse disciplines, including linguistics, artificial intelligence, and computer science, with the primary aim of enabling machines to engage with human language in a meaningful manner. Consequently, NLP algorithms may be used to extract information from unstructured texts (Singh, 2018). Despite the success of DL models in ecology using tabular, image, and acoustic data, few studies have explored the potential of NLP models for ecological data analysis and interpretation (Farrell et al., 2022). So far, most modeling approaches have been applied to detect trends and perform evidence synthesis and literature reviews (Farrell et al., 2022). For example, NLP has been used to identify whether scientific articles are relevant to ecological databases such as the Living Planet Database (http://livingplanetindex.org/data_portal) or the PREDICTS database (Hudson et al., 2014), helping to accelerate dataset construction by reducing manual screening (Cornford et al., 2021). Another application of NLP in ecology is to automatically extract taxonomic name information from ecological documents using named entity recognition DL models (e.g., TaxoNERD; Le Guillarme and Thuiller, 2022). Similarly, NLP has previously been used to identify and extract functional trait information from unstructured texts (Endara et al., 2018; Mora and Araya, 2018; Folk et al., 2024). However, these approaches have employed simple models such as dictionaries, term co‐occurrences, and bag‐of‐words (BOW) and rule‐based statistical models, which have disadvantages in terms of complexity and predictive power compared to modern large language models (Min et al., 2023).

Here, we propose a pipeline of NLP techniques to automatically extract functional trait data from unstructured texts including the processes of data acquisition, preprocessing, and model selection and validation. We formulate the problem of trait value prediction as two standard NLP tasks: (1) classification for categorical traits and (2) question answering for numerical traits. Using two sources of textual species descriptions, we train and evaluate a range of models, from a straightforward keyword search to state‐of‐the‐art large language models, and compare their performance.

## METHODS

### Task formulation

**Figure 1:**
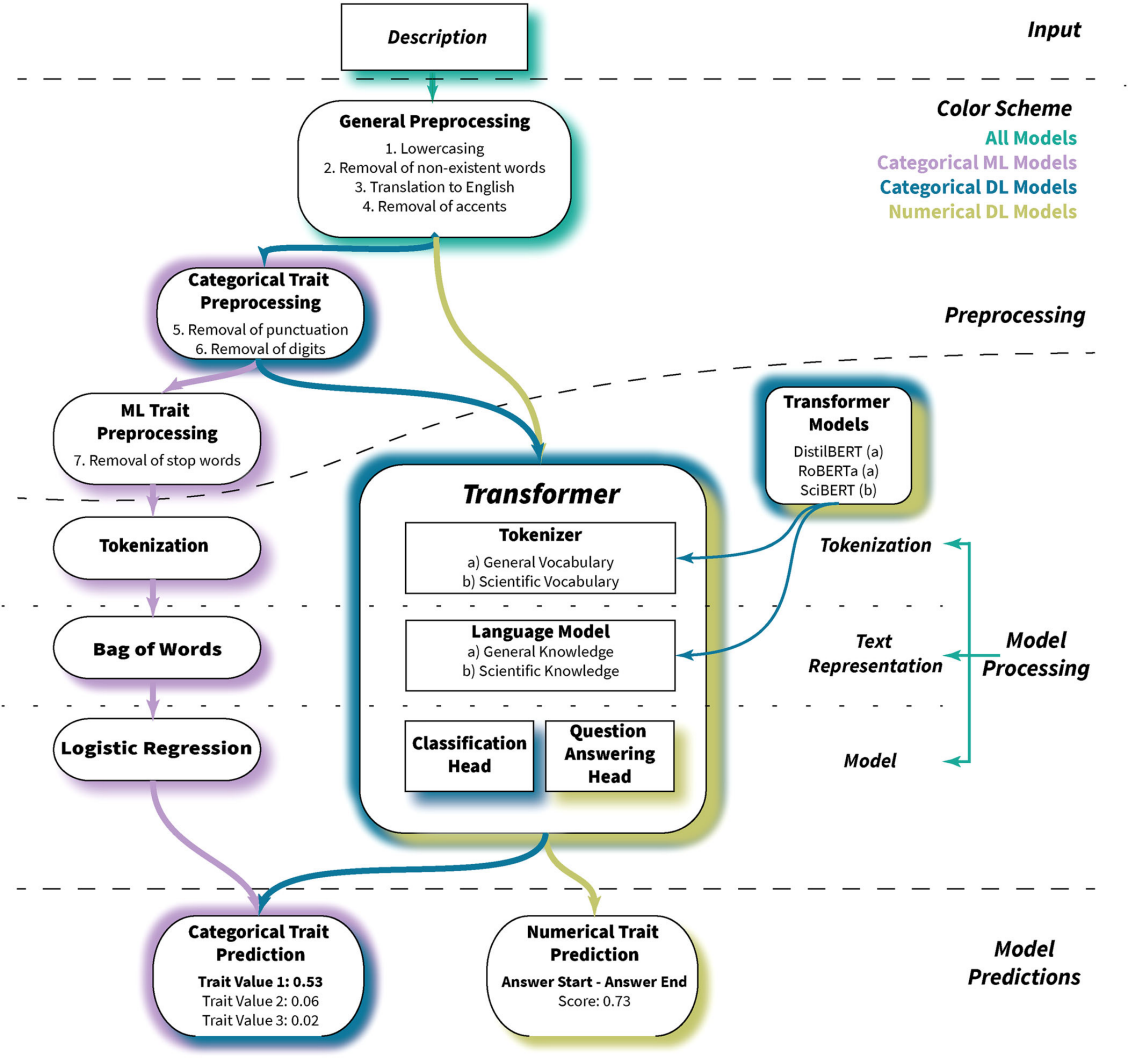
The NLP pipeline used for the prediction of categorical and numerical traits based on textual species descriptions. The description first goes through a preprocessing pipeline and then enters the categorical machine learning (ML) pipeline (violet), categorical deep learning (DL) pipeline (blue), and numerical DL pipeline (green). Within the corresponding pipelines, the description is further preprocessed if necessary and then tokenized and transformed into a vector embedding. Finally, the vector embedding is put into the corresponding model head and the model returns a prediction with an associated confidence score. An example description being processed using the pipeline is presented in Appendix S1.

The proposed pipeline to extract functional traits from text starts from a textual description of a species. This description can be taken from a variety of sources, including floras, scientific papers, and plant databases, and usually contains species trait information for a few to a dozen traits. Following standard NLP preprocessing, the description is then used as the input for ML or DL models. The output of the models is a predicted trait value with a corresponding confidence score. We use two different supervised NLP tasks for categorical and numerical traits, respectively. The pipeline (Figure 1) is described in detail below.

#### Categorical traits: Sequence classification

Categorical traits have a discrete number of possible values. We considered five such traits, as defined in the GIFT database (Weigelt et al., 2020), representing important structural and life history aspects of plants (Taylor et al., 2023). The five traits are: (1) growth form: herb, shrub, tree (combinations possible); (2) epiphyte: epiphyte, terrestrial; (3) climber: climber, self‐supporting; (4) life cycle: annual, perennial (combinations possible); and (5) life form: phanerophyte, chamaephyte, hemicryptophyte, cryptophyte, therophyte (combinations possible). We slightly modified some trait definitions (e.g., simplified the epiphytism and climbing habit traits by removing the facultative value) and removed the biennial life cycle, due to the very low representation in the data (less than 1%). Including the biennial life cycle would have resulted in a very imbalanced dataset that would require a different set of tools (e.g., few‐shot learning) (Wang et al., 2020).

Given the finite number of classes per trait, predicting a categorical trait value can be considered a sequence classification task. Unlike keyword search algorithms, which rely on manually defined rules to predict the trait value, ML and DL models automatically assign weights to words or phrases that are relevant to the trait value and are able to make predictions without explicit mentions of trait values. We trained a multi‐class classification model for each trait, from which a probability is assigned for each trait value. Using these probabilities, there are two ways to obtain a prediction. The first method, which we used as the default for our models, is to select the trait value with the highest probability. However, this approach has two limitations: it can only predict one trait value, which precludes the use of combinations, and it may provide a prediction even if the probability of all traits is low. The second way to obtain the prediction is to predict all trait values with a probability above a defined threshold. This allows combinations of trait values (e.g., “herb/shrub”) if the probabilities of both trait values are above the threshold. Additionally, if there is insufficient support for a trait, the probabilities should be below the threshold and therefore no value will be predicted.

#### Numerical traits: Extractive question answering

Numerical traits are those described by a continuous numeric variable. Here, we considered three such traits that are commonly reported in species descriptions and encapsulate important information on plant functional strategy (Wright et al., 2004; Díaz et al., 2016): (1) maximum plant height (in meters); (2) maximum leaf length (in centimeters); and (3) maximum leaf width (in centimeters).

The sequence classification approach outlined above cannot be used for numerical traits due to the infinite range of possible values. Consequently, traditional ML models, such as logistic regression, cannot be employed for this task and instead large language models must be used. This led us to adopt an extractive, or contextual, question‐answering (QA) model to constrain the numerical trait prediction within the text. Extractive QA models ask a question (e.g., What is the height of the plant?) and return an answer and confidence score based on a context paragraph (in our case, the species description). Due to the nature of the model, it returns the unit along with the numerical value, making it possible to convert between units. To obtain the final predicted number, we post‐processed the answer from the QA model by filtering out answers that did not contain a number and a unit. We further excluded predictions with a confidence score below a defined threshold. If the answer contained two numbers (e.g., “perennial herb, 15–40 cm tall”), we extracted the second value, under the assumption that they represented a range (minimum–maximum), based on patterns we observed in our datasets; this value was then transformed into the required unit of measurement for the trait.

### Data

All of the above‐defined problems are supervised NLP tasks and thus require textual data and corresponding labels. For this reason, we performed a web scrape to acquire species descriptions from two large online plant knowledge bases: Plants of the World Online (POWO; http://www.plantsoftheworldonline.org/), which is based on information from regional floras, and English‐language plant articles on Wikipedia (WIKI; https://en.wikipedia.org/), which are based on information written by volunteers. We divided the descriptions from these databases by species and by source and then used them as the textual input in the NLP models.

Data from POWO includes information on the taxonomy and, most importantly for our study, textual descriptions of traits, identification, and distribution information at the species level. The descriptions are categorized hierarchically, containing information on various characteristics such as leaf morphology, plant habit, and reproductive information. We used the taxize R package (Chamberlain et al., 2020) to retrieve species descriptions from POWO, resulting in 288,254 descriptions for 59,151 plant species in 251 distinct categories containing information on morphology, habitat, and distribution, each presented at multiple levels of detail. Using only descriptions from a category that contains information on a particular trait may improve model performance compared to using additional less relevant data that may lead to erroneous correlations. To test this, we created three separate datasets: one using the entire POWO knowledge base, and two trait‐specific datasets that use only the descriptions from certain categories. The POWO dataset was built by combining all descriptions per species per source and is the main one we used in the current study. The POWO_MGH dataset uses only descriptions from the morphology general habit category containing relevant information for the traits growth form, epiphyte, climber, life cycle, and plant height. Similarly, the POWO_ML dataset solely uses the morphology leaf category that includes data on leaf length and width. Further information on the POWO dataset can be found in Appendix S2.

To retrieve species descriptions from Wikipedia, we searched for English‐language articles of the ~270,000 species for which we found information for at least one of the selected functional traits in the GIFT database. We used the Python Wikipedia‐API (https://pypi.org/project/Wikipedia-API) and the Requests (https://pypi.org/project/requests/) and Beautiful Soup (https://pypi.org/project/beautifulsoup4/) web scraping libraries. This resulted in 194,994 descriptions for 55,631 species with description categories based on the sections in Wikipedia. Further information on the Wikipedia dataset can be found in Appendix S2.

The trait values that we used to train and evaluate the models were extracted from the GIFT database (Weigelt et al., 2020), which is a global archive of regional plant checklists, regional floras, and plant functional traits containing information for 109 traits for more than 290,000 species. Using the GIFT R package (Denelle et al., 2023), we extracted the values for the traits of interest from GIFT version 3.0. We then merged descriptions and traits by species name, aligning them according to the taxonomic nomenclature from the World Checklist of Vascular Plants (Govaerts, 2024), and used these traits as labels in the supervised learning tasks, following a distant supervision approach (Go et al., 2009; Smirnova and Cudré‐Mauroux, 2018). It should be emphasized that not all traits have the same coverage in GIFT, which leads to significant differences in the number of labeled samples per trait (Tables S1 and S2 in Appendix S2).

### Data preprocessing

Preprocessing for the POWO and WIKI datasets was performed using the NLTK Python library (Loper and Bird, 2002) and varied slightly for the categorical ML, categorical DL, and numerical DL models due to the different input requirements of the models. The base preprocessing pipeline for all models consisted of the removal of artifacts and accents from the text. Using a keyword search that focused on language‐specific terms, we detected 20,034 non‐English descriptions in the POWO dataset out of the total 288,254 descriptions, 19,945 of which were Spanish. These descriptions were then translated using the GoogleTranslate Python API (https://pypi.org/project/googletrans/). The text was lowercased and split into tokens, which in our case may represent words, numbers, or punctuation. For the categorical models, we additionally removed all digits and punctuation as they are not informative for this type of analysis. Finally, for the categorical ML models, we also removed English stop words (e.g., “the”) as they do not provide any trait information.

### Models

#### Keyword search

We compared the performance of the categorical models to a simple keyword search, a commonly used technique in the automated extraction of traits (Coleman et al., 2023). To this end, we created a dictionary for each trait and used a script based on regular expressions to classify the descriptions. The keywords were the trait value, any synonyms that might be found in the description, as well as trait–trait relations (e.g., a therophyte [life form] being an herb [growth form]). A full list of the keywords used can be found in Appendix S3.

#### Logistic regression

As a baseline for the categorical ML modeling approach, we chose logistic regression, a parametric predictive classification model, due to its wide use in ecological research. The model assumes a linear relationship between the independent variables and the target variable and predicts a probability for each class. However, as the model requires numeric input, we first transformed the textual input to a numeric vector space to create a text representation. To this end, we used the BOW model, also known as one‐hot encoding. The BOW model takes the most common words (in this case, the top 1000) in the entire textual corpus to form the vocabulary of the model (Zhang et al., 2010). Each description is then transformed into a vector of size 1000, whose values correspond to the number of times each term appears in the description. The BOW representation vector is used as the predictor in the logistic regression model, while the trait value is the outcome. We implemented the BOW and logistic regression models using the scikit‐learn ML library (Pedregosa et al., 2011) (Appendix S4).

#### Transformers

Transformers (Vaswani et al., 2017) are the basis of large language models that have recently revolutionized NLP, as well as other fields including computer vision and digital signal processing, sparking a revolution in how DL models are built. Transfer learning allows knowledge the transformer has acquired from one task or dataset to be applied to another task in a similar context. In addition, an attention mechanism enables the model to learn long‐range dependencies without the use of recurrent layers, as in earlier models, allowing the models to achieve state‐of‐the‐art performance on a variety of NLP tasks.

Encoder models, like Google's BERT (Devlin et al., 2018), are a class of transformers characterized as having “bi‐directional” attention, because at each stage the attention layers can access all words in the sentence. This allows encoder models to excel at tasks where an understanding of the entire textual input is required (e.g., sequence classification and extractive QA), making them the go‐to architecture for the type of questions being addressed here. Encoder models consist of three core components: (1) the tokenizer learns the vocabulary of the model and converts the input text into machine‐readable data; (2) the language model includes the attention layers and aims to learn a vector representation of the text that encodes the meaning of words and how they co‐occur (e.g., words with some connection, such as “Japan” and “sushi,” are closer together in the embedding space compared to words like “Japan” and “pizza”; Mikolov et al., 2013); and (3) the task‐specific head, through the method of fine‐tuning, enables the model to learn task‐specific features while retaining the general language knowledge acquired during pre‐training. We use a sequence classification head for categorical traits and an extractive QA head for the numerical traits.

We trained and evaluated the following three models using the Huggingface's transformers Python library (Wolf et al., 2019) (Appendix S4):

1. *DistilBERT*: The DistilBERT model (Sanh et al., 2019) is trained in a self‐supervised fashion using the BERT model as a teacher, a process called knowledge distillation. This allows the model to achieve results comparable to BERT with less than 20% of the parameters (66 million compared to the 340 million of BERT), making the model much easier to train and use. More specifically, it is pre‐trained using masked language modeling, where a part of a sentence is masked out and the model is trained to predict it. Furthermore, it is pre‐trained with a distillation loss and cosine embedding loss, such that the prediction probabilities and hidden states of the model are as close as possible to those of BERT. The texts that this model was trained on are the same as for BERT, a corpus comprising the Toronto Book Corpus and the English Wikipedia Corpus; therefore, the vocabulary and language model of DistilBERT are general knowledge related. To use this model in our categorical trait pipeline, we attached a sequence classification head and fine‐tuned it using our species descriptions and trait data. For our numerical trait pipeline, we attached a QA head that had been fine‐tuned on the SQuAD v1.1 dataset (Rajpurkar et al., 2016), which contains more than 100,000 question–answer pairs in a variety of contexts.
2. *SciBERT*: SciBERT (Beltagy et al., 2019) uses the same architecture as BERT but is trained on papers from the corpus of Semantic Scholar (https://www.semanticscholar.org/), consisting of 1.14 million papers and 3.1 billion tokens. Consequently, SciBERT has a vocabulary (scivocab) built to best match the training corpus, meaning that the language model and vocabulary are focused on scientific knowledge. We used this model only for the categorical traits by fine‐tuning it identically to DistilBERT.
3. *RoBERTa*: The RoBERTa model (Liu et al., 2019) optimizes BERT's pre‐training process using only the masked language modeling objective. For the extraction of numerical traits, we used a version of RoBERTa with a QA head. The QA head had been fine‐tuned on the SQuAD v2.0 dataset (Rajpurkar et al., 2018), which builds on the first version of the dataset by adding unanswerable questions. This should theoretically improve the model's ability to identify cases where no numerical traits exist in the description.

### Evaluation

We split the textual descriptions and trait data into a training set (75% of the data) and a test set (25%). Because of the large amount of data, we only used one split instead of a cross‐validation approach. We evaluated the categorical traits using the following metrics that are commonly used to quantify the performance of classification models:

Accuracy: The number of descriptions correctly classified into a trait value scaled by the total number of descriptions.

$$\mathrm{Accuracy}=\frac{\#\mathrm{true positives}+\#\mathrm{true negatives}}{\#\;\mathrm{samples}}$$

Precision: The proportion of descriptions correctly classified into a given trait value scaled by the total number of descriptions classified into a given trait value.

$$\begin{array}{l} Precision\;=\;\frac{1}{\#\;classes}\sum_{class}\frac{\#truepositives_{class}}{\#truepositives_{class}+\#falsepositives_{class}} \end{array}$$

Recall: The proportion of descriptions correctly classified into a given trait value scaled by the total number of descriptions that are labeled as a given trait value in the dataset.

$$\mathrm{Recall}=\frac{1}{\#\;\mathrm{classes}}\sum_{\mathrm{class}}\frac{\#\mathrm{true positive}s_{\mathrm{class}}}{\#\mathrm{true positive}s_{\mathrm{class}}+\#\mathrm{true negative}s_{\mathrm{class}}}$$

F1 score: The harmonic mean of precision and recall.

$$\text{F1}\;\mathrm{score}=\;\frac{1}{\mathrm{\# classes}}\sum_{\mathrm{class}}\frac{2*\mathrm{Precisio}n_{\mathrm{class}}*\mathrm{Recal}l_{\mathrm{class}}}{\mathrm{Precisio}n_{\mathrm{class}}+\mathrm{Recal}l_{\mathrm{class}}}$$

While accuracy is the most popular metric, it can be unrealistically high in imbalanced datasets such as ours, where the number of descriptions per trait value is not uniformly distributed; therefore, we primarily focused on the remaining metrics. As precision, recall, and F1 score are calculated for each class separately, we worked with the macro‐version of the metric, meaning that we averaged over all trait values per trait. To evaluate how well the models performed on datasets with distinct characteristics from those used for training, we carried out an inter‐dataset evaluation, where models trained using one dataset's training set were evaluated on the test sets of the remaining two datasets (Appendix S5). Furthermore, when using the probabilistic output of the models combined with a threshold *t*, the probability threshold can be increased or decreased to achieve results with higher precision or higher recall, respectively. To analyze the model behavior dependent on the probability threshold, we calculated receiver operating characteristic and precision–recall curves, which show the precision versus recall at a variety of given thresholds. Furthermore, we analyzed the model performance when using a threshold of *t* = 0.5 and *t* = 0.8 and compared it to our initial results (Appendix S6).

To evaluate the numerical trait models, we first log‐transformed the trait values due to their skewed distributions. To allow for the comparison of the results between the different datasets and models, the data were normalized to a range between 0 and 1, and we used the normalized mean absolute error (nMAE) as the main metric of model performance. Furthermore, we calculated the coverage, which represents the proportion of answers above the threshold that resulted in a trait value and unit of measurement. We evaluated the models on the aggregate POWO dataset and compared this to the trait‐specific POWO_MGH and POWO_ML datasets to assess the difference between using a smaller dataset versus one including entire descriptions, which could include significant amounts of interfering information. The threshold of the numerical models was set to *t* = 0.5 for DistilBERT and to *t* = 0.25 for RoBERTa. We used a lower threshold for RoBERTa as it is trained on the SQuAD v2.0 dataset which includes unanswerable questions, thus the model prediction scores should skew towards a score of 0 for descriptions with no trait information.

## RESULTS

### Categorical traits

**Figure 2:**
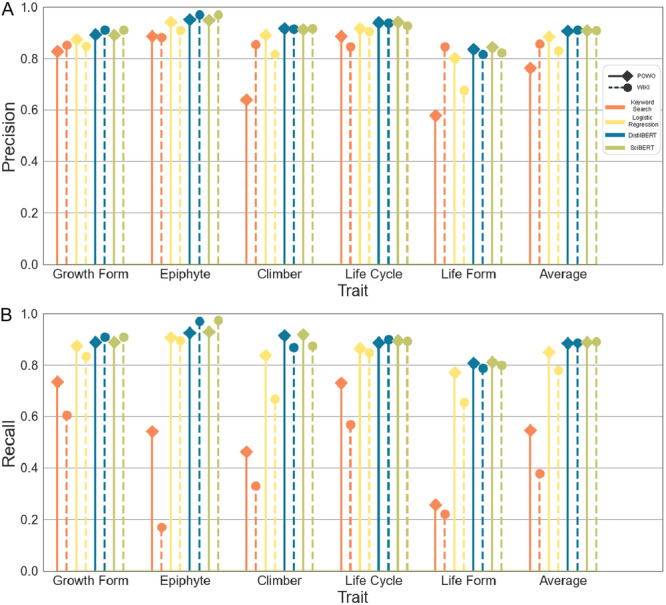
Model comparison across the Plants of the World Online (solid line diamond) and Wikipedia datasets (dashed line circle). Precision (A) and recall (B) for the categorical traits are shown for the keyword search (orange), logistic regression (yellow), DistilBERT (blue), and SciBERT (green).

The transformer models outperformed the standard keyword search model and the logistic regression model. On the POWO dataset, DistilBERT achieved an average precision across all traits of 90.6% and a recall of 88.5%. In comparison, the logistic regression model achieved an average precision of 88.4% and a recall of 85.1%, while the keyword search model had an average precision of 76.4% and an average recall of 54.6% (Figure 2). The performance of SciBERT was comparable to that of DistilBERT, with an average improvement of only 0.2% across metrics. On the WIKI dataset, the keyword search had significantly fewer predictions, with a recall of 37.9%, meaning that a large amount of these descriptions did not contain information on the trait. Similarly, as the WIKI dataset contains a much smaller amount of expert information that directly includes trait information, the performance of the logistic regression model decreased by an average of 5% compared with the POWO dataset, reporting a precision of 83% and recall of 80%. The performance of the transformers that consider not only the occurrence of individual keywords but also the broader context was not affected by this factor, and the scores of both models increased slightly, with a precision of 91% and recall of 88.7% for the DistilBERT model, and a precision of 91% and recall of 89% for the SciBERT model.

**Figure 3:**
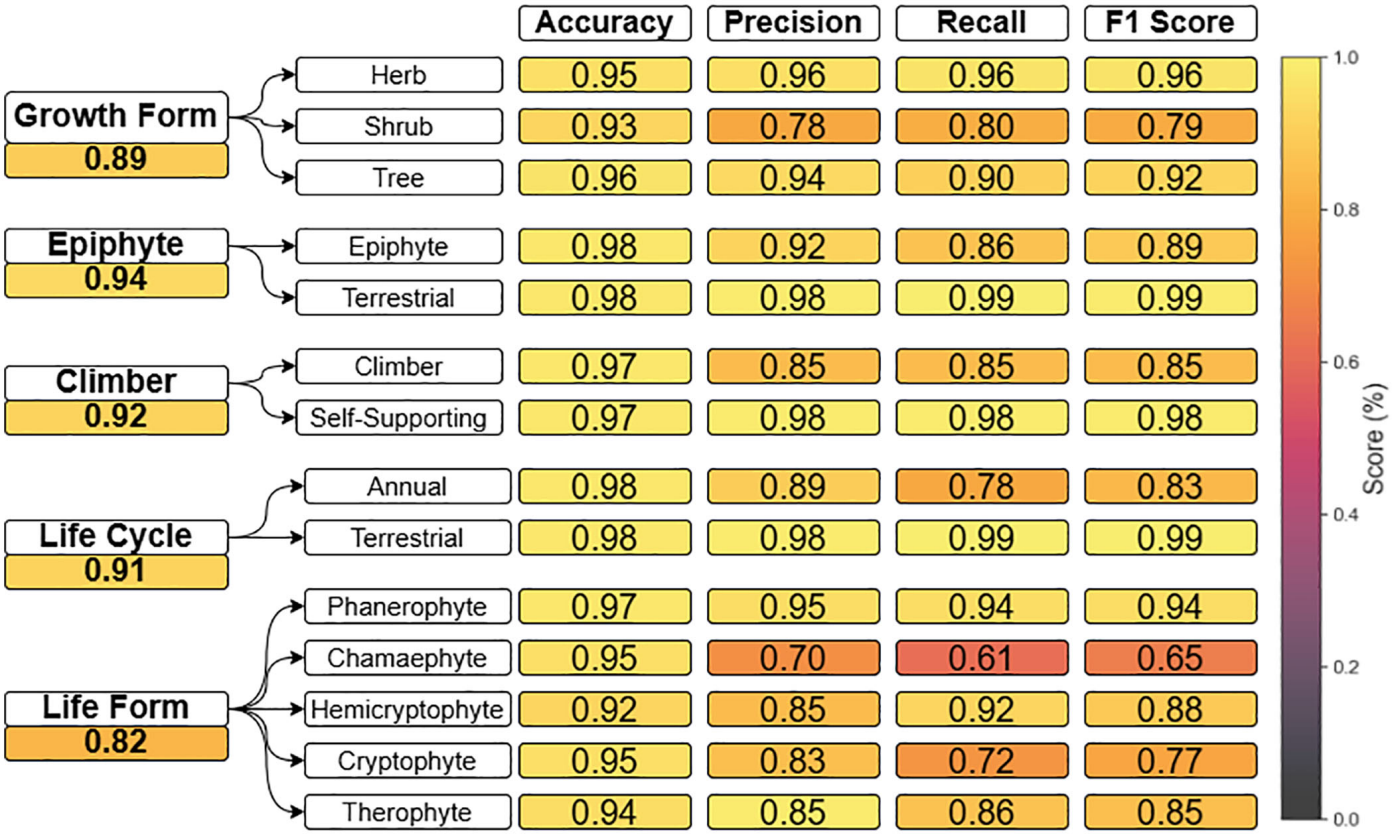
Class‐specific performance metrics for each categorical trait value on the Plants of the World Online dataset using the DistilBERT model. The macro F1 score of the trait (average across trait values) is shown below the trait name.

While both DistilBERT and SciBERT showed the best results, we chose to focus on DistilBERT for the rest of the analyses due to its smaller number of parameters. The model performed best on the epiphyte trait (F1 score of 93.8% and 97%) and worst on the life form trait (F1 score of 82% and 79.7%) on the POWO and WIKI databases, respectively. This variation in performance was also seen among traits (Figure 3). For instance, within the binary traits (i.e., epiphyte, climber, and life cycle), there was an approximately 13% decrease in F1 score for the class with a lower representation in the data. In the growth form trait, the shrub class had a similar decrease in F1 score (15%) compared to the other two growth forms. The largest variation was seen in the life form trait, where class F1 scores ranged from 65% (chamaephyte) to 94% (phanerophyte).

### Numerical traits

**Figure 4:**
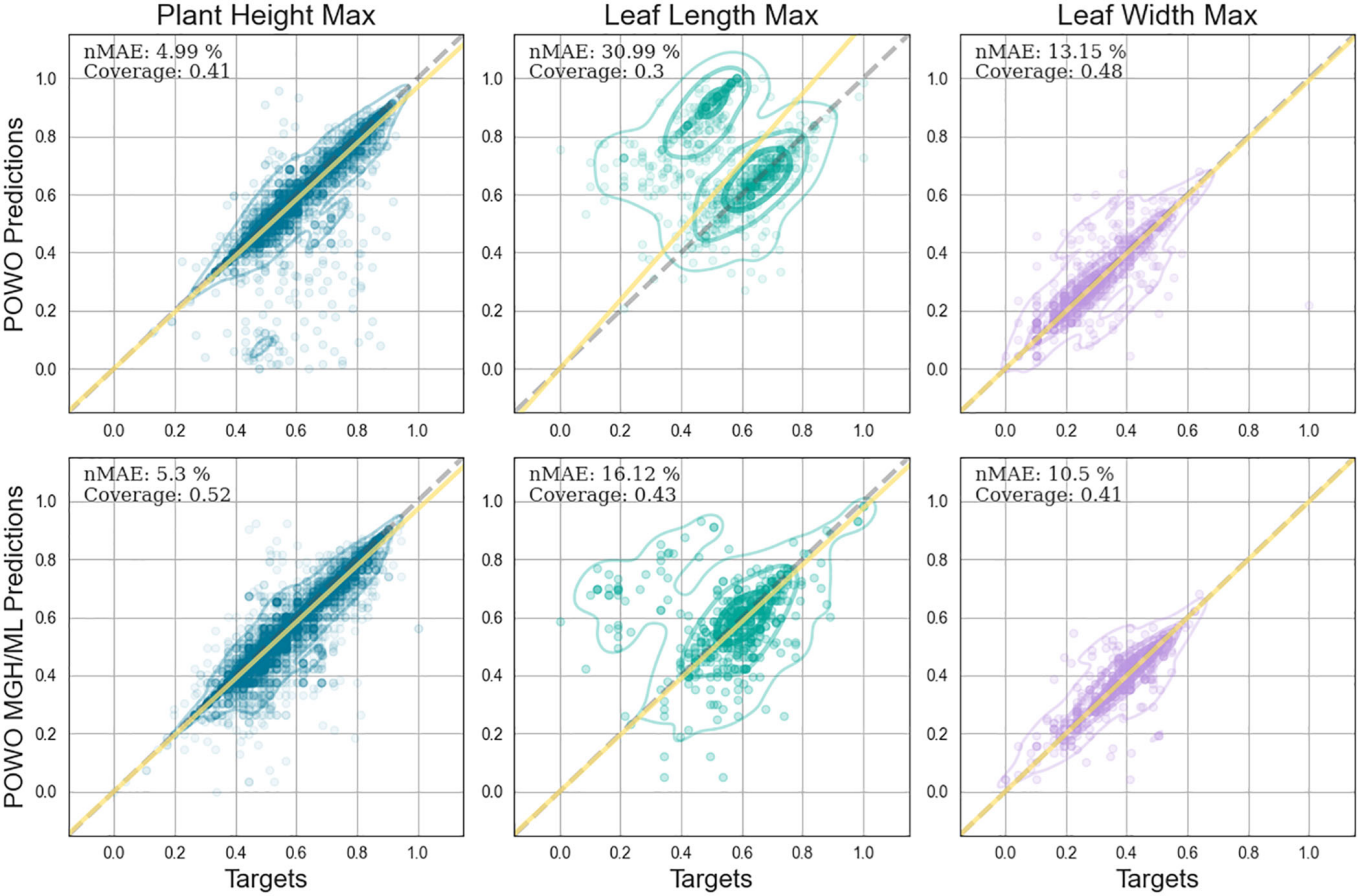
Observed versus predicted numerical traits for the RoBERTa model on the aggregated Plants of the World Online (POWO) and trait‐specific POWO datasets (descriptions utilizing only specific POWO categories: MGH, Morphology_General_Habit; ML, Morphology_Leaf). The numerical traits are represented as plant height (blue), leaf length (cyan), and leaf width (violet). The 95% and 50% kernel density estimates are also shown as polygons in the corresponding trait color. The 1:1 line (gray dashed) and the regression line between the observed and predicted values (yellow solid) are also shown. nMAE, normalized mean absolute error.

Depending on the trait of interest and the database in question, 82% to 100% of the original descriptions contained a numeric value and a unit of measurement and were subsequently used as input in the QA models. The predictions on the trait‐specific POWO datasets resulted in a higher coverage of answers (50.83%) compared to the entire POWO dataset (39.17%), likely because more descriptions from the entire POWO dataset do not contain information on the trait of interest. Consequently, the nMAE across all traits was also significantly decreased in the trait‐specific datasets, with an average decrease of 5.74% for RoBERTa (Figure 4) and 7.32% for DistilBERT (Figure S10 in Appendix S8).

RoBERTa achieved the smallest nMAE (10.64%) averaged across all traits on the trait‐specific datasets. However, this varied significantly between traits, with a value of 5.3% for plant height and 16.12% for leaf length. This pattern was consistent across models and datasets, with the lowest errors for plant height (averaging 5.68%) and the highest for leaf length (averaging 24.39%). DistilBERT performed slightly worse than RoBERTa, with an average nMAE of 13.1% on the trait‐specific datasets. DistilBERT's coverage was 11% higher than that of RoBERTa, despite the smaller probability threshold used for RoBERTa (*t* = 0.25); however, as we were using descriptions and trait data from different sources, this increase could be due to either the higher recall of the model or to false positive extractions. Furthermore, the more compact architecture of DistilBERT led to a several‐fold acceleration in the QA process, which might be relevant for some tasks.

## DISCUSSION

Overall, the NLP pipeline using large language models to extract categorical plant traits from texts achieved high average precision (≥90.6%) and recall (≥88.5%) across all traits, outperforming typically used keyword search models. Similarly, the pipeline for numerical traits using RoBERTa achieved small errors across all traits (averaged nMAE = 10.64%). Consequently, the NLP workflow we describe here has the potential to streamline the digitization and extraction of plant functional traits from textual descriptions. At a rate of 10 seconds per trait, manual trait extraction for the eight selected traits from the 114,782 descriptions in the POWO and WIKI datasets would take approximately 1.2 years of 40 working hours per week. In contrast, the proposed workflow can extract the traits in a few minutes to hours without human supervision, saving time and resources. Because the model expects descriptions to be associated with a specific species, when employed in less structured use cases than ours, it should be combined with other tools such as optical character recognition and the recognition of taxa and their corresponding descriptions (Le Guillarme and Thuiller, 2022; Folk et al., 2024). These complementary NLP tools may enhance the input data fed to the large language models, thereby improving the comprehensiveness of the input as well as allowing the use of a broader scope of textual data. As a result, an enormous amount of information currently hidden in unstructured texts online, in regional floras and in other scientific publications, can be made digitally accessible for functional plant ecology studies.

A few points should be considered when using the pipeline and the NLP models for trait extraction from texts. As can be seen in our results, the performance of the models is dependent on several factors, the first of which is the type of data the model is trained on, such as whether it is an expert resource, like the POWO dataset, or a more general resource, like the WIKI dataset. Furthermore, the output of the models cannot be taken for granted. Similar to other applications of DL in ecology (e.g., animal detection in camera traps; Norouzzadeh et al., 2018), the goal is to allow for more efficient processing of large amounts of data. There is, however, a tradeoff between the amount of predicted data and precision, which the user can actively manage. The probabilistic output of the model allows users to set the probability threshold high if they require a smaller number of predictions with high precision, or to set the threshold low to obtain a higher recall but a lower reliability of the data requiring a longer time for manual processing. Finally, large language models can derive trait information from the contextual cues in a text (including potential trait–trait correlations and phylogenetic relationships), allowing them to potentially identify values for traits that might not be explicitly mentioned. While the capacity of the model to “read between the lines” can be particularly useful when dealing with ecological literature that may not always provide detailed or standardized descriptions of traits, it also means that the model can occasionally generate incorrect or unfounded predictions.

For the categorical traits, SciBERT had a slightly better performance compared to DistilBERT, with an average improvement of 0.2%. This could be attributed to the scientific vocabulary and language model in SciBERT, which is relevant given that the POWO dataset includes a large amount of technical terminology. Alternatively, the increase in performance may be due to the higher number of parameters in the SciBERT model. The categorical model performance varied across traits, with F1 scores ranging between 79.7% and 97% for the different traits and datasets. These differences can be explained by a few factors. One is whether the trait information is frequently included in the descriptions. While traits such as growth form are commonly reported in the descriptions we use, either explicitly or implicitly, other traits (e.g., life form) had less than 1% explicit coverage in both datasets and a small number of implicit references, making them much more ambiguous to detect. A second factor is the difficulty of the trait assignment. While some trait values can be determined based on simpler rules that the models can discover (e.g., annual → herb), classification of other trait values (e.g., cryptophyte) requires either explicit or a large amount of implicit information. The final factor that may influence the results is the variability of the trait itself within the literature. While the epiphytism and life cycle traits have rather consistent values across sources, the boundaries between growth forms like “herb” and “shrub” or “shrub” and “tree” are not as clearly defined and can vary according to the source. These factors also resulted in a variability in the model performance among traits. For instance, the 15% decrease in F1 score for the shrub growth form was due to there often being no clear delineation between an herb and a shrub and even less so between a shrub and a tree. For example, some species (e.g., grass trees, tree ferns, semi‐woody megaherbs or dwarf shrubs, bamboos, palms, climbers) are difficult to classify into the standard growth form categories—both for ecologists and for artificial intelligence—and consequently their treatment often differs among resources (Wenk et al., 2024). In some descriptions, a species is described as an herb, while in the GIFT data it is labeled as a shrub. Therefore, the variations in model performance could partly be attributed to the discrepancy between the description and label data, rather than to the model's inference capabilities. The trait for which our results showed the largest discrepancies, life form, suffered from the first two problems—the lack of information in the descriptions and the difficulty of assessment. This was not an issue for trait values like phanerophyte, which has an F1 score of 94%, as the model can discover the relationship indicating that species with a tree growth form fall under the phanerophyte life form, while other trait values (e.g., chamaephyte) had significantly lower scores (65%). Similar variation can be seen between the numerical traits. For example, plant height has a thorough coverage in texts and is usually clearly specified, whereas leaf length and width are not as well represented and, even when they are, abbreviations are commonly used and information can be specified separately for the lamina, including or excluding the petiole, leaflets, and other structures. All of these factors increase the difficulty of the QA task and result in higher errors for the leaf traits.

Another important aspect to consider when interpreting the performance of the NLP pipeline is the fusion of data from two different sources. Because we evaluated the traits in GIFT, an external database, using distant supervision instead of labels extracted directly from the descriptions, mismatches may arise. As a result, an optimal accuracy of 100% and an nMAE of 0% is not likely even in the case of a human extracting traits. For the classification models for categorical traits, this is due to inconsistencies in the trait values extracted from different sources as well as the inherent difficulty of specifying the class of some species' traits as outlined above. To assess this, we can use the agreement score available per trait in the GIFT database (Weigelt et al., 2020), which represents the agreement of different sources for a trait value per species. For the species in the POWO dataset, the average agreement score for growth form in GIFT is 92.2%, with an average of 4.06 descriptions per species. In other words, if we use the primary label in GIFT to label all of the 199,241 descriptions, we get an accuracy of 92.2%. In comparison, our model has an accuracy of 92.1%, meaning that it is sufficiently high given the circumstances. For the other categorical traits, the accuracies based on the GIFT agreement scores are 99.5% for epiphytism, 93.3% for climbing habit, 98.1% for life cycle, and 97% for life form. The corresponding DistilBERT accuracies were 97.9% for epiphytism, 97.1% for climbing habit, 97.9% for life cycle, and 86.3% for life form. Similarly, numerical traits vary over time and space, and different floras might report different values, thus there is an inherent variation in the results reported in the literature. In GIFT, plants described in multiple sources have an average coefficient of variation of 0.48 for plant height, 8.43 for leaf length, and 0.59 for leaf width. Furthermore, data on traits can be reported in a variety of ways. For example, the size of a plant can be reported as stem length, vegetative or reproductive height, growing height of a vine, length of a shoot of a creeping plant, and so on, making it difficult to arrive at a conclusive plant height estimate. Some resources report an average mature height, while others report a rarely achieved maximum growing height. Leaf size can be reported as lamina dimensions or blade dimensions including the petiole; for compound leaves, these dimensions may refer to the leaf or leaflets (e.g., see Figure 4 for leaf length). Upon a manual examination of the differences between predicted and label data, we saw that some of the seemingly incorrect predictions can be attributed to species with compound leaves, and while the model extracts the size of the leaf blade, some of the data in GIFT may be associated with the size of the leaflets. This variation in reporting makes it difficult to label even when manually processing the data (Kattge et al., 2020; Maitner et al., 2023; Wenk et al., 2024).

Another potential problem of our approach is the overconfidence of the model when provided with limited information. The training approach is based on distant supervision, resulting in noisy labels. When trait information is missing in the description, the model may learn erroneous associations, which can introduce two key issues related to low data coverage. First, some traits have very little explicit information available, leading the model to infer values based on indirect associations, such as co‐occurring traits or even locations and habitats. A possible way to help the model distinguish between informative and uninformative descriptions in the future is to add negative descriptions within the training process, which contain no relevant trait information and are labeled accordingly, as well as other methods of noise reduction found in the distant supervision literature (Smirnova and Cudré‐Mauroux, 2018). Second, certain traits have a “default” state that is rarely mentioned in descriptions but implicitly assumed, such as terrestrial growth form in plants. Our model demonstrates the ability to infer such implicit traits in the absence of explicit mentions, although this capability might introduce biases or unintended patterns. While the approach of fusing textual data and target descriptions from independent resources has its problems, it also has many advantages, the most important one being the ease of adding a large amount of data from different resources (Go et al., 2009). This allows the model to be easily expanded to new traits, descriptions, and even languages in a semi‐automated manner, without the need for significant human effort.

An alternative approach to our problem is to frame the trait extraction as a named entity recognition (NER) task, a method that has been successfully utilized for phenotype extraction in the wheat breeding literature (Nédellec et al., 2024). However, we propose several advantages to our approach. For categorical traits, utilizing an NER approach would limit the trait extraction to traits that are explicitly mentioned in the text, functioning similarly to an intelligent keyword search. Because of this limitation, the NER approach is unable to capture any indirect references, nuances, or implied information about traits from the surrounding context. For the numerical traits, a QA approach can be preferable to NER as it enables the targeted retrieval of specific values in context, accommodating multiple levels of granularity (e.g., distinguishing between “leaf length” and “lamina length”) and ensuring that the extracted measurements are relevant to the precise trait of interest.

There are several ways to further improve the capabilities of the model. One step is to expand the use of the model to languages other than English, rather than using translations as was done in our workflow. Because many regional floras are written in the primary language of that country, this approach can expand the scope of included traits, which are generally biased towards the Global North and Australia. This can be done either by fine‐tuning a multilingual language model, such as the multilingual version of BERT or other architectures like BLOOM (Le Scao et al., 2022), or by fine‐tuning monolingual language models like Spanish‐BERT (Cañete et al., 2023), GottBERT (Scheible et al., 2020), and AraBERT (Antoun et al., 2020). Another step is to adapt the domain of the transformer models (Gururangan et al., 2020) to ecological tasks, similar to what has been done in other fields (e.g., BioBERT for biomedicine [Lee et al., 2020] and FinBERT for financial analysis [Liu et al., 2021]). This should theoretically improve the model performance on a variety of tasks related to ecology, including functional trait extraction. A foundation model for plant ecology could be used in the current pipeline, as well as for tasks including literature reviews, text summarization, and named entity recognition. The field of NLP information extraction is rapidly evolving, with generative artificial intelligence models opening new possibilities for improving accuracy and scalability, including specialized models like *text‐bison*, which demonstrate the potential for reliable automated extraction (Gougherty and Clipp, 2024). Finally, the text processing pipeline can be combined with other information and data modalities. The inclusion of geographic or phylogenetic information in the form of a prior has already resulted in a large improvement in classification performance in species identification from images (Mac Aodha et al., 2019). Including trait imputation methods, i.e., modeling trait values based on trait–trait correlations and phylogenetic information, in the pipeline would potentially improve the results (Joswig et al., 2023). As the success of imputation increases with the availability of more traits per species, the higher taxonomic, geographic, and functional coverage of the traits predicted using the NLP pipeline would facilitate more accurate predictions for the remaining traits. Furthermore, trait imputation methods could be used to flag suspicious or implausible trait values resulting from the NLP pipeline. Therefore, combining this information with other data, such as images from the entire plant or plant parts, will further increase the capabilities of the model and result in more reliable predictions.

Overall, the NLP workflow presented here holds great potential to overcome resource limitations in mobilizing plant functional trait data and may promote a more comprehensive and global understanding of functional plant ecology. While most landmark publications in functional macroecology have so far been based on trait data for up to a few thousand species and include strong taxonomic and geographical biases (Maitner et al., 2023), truly global and less biased analyses now seem to be within reach. There is a huge amount of species descriptions and trait information readily available in scientific papers, preprints, online libraries (e.g., Biodiversity Heritage Library), thematic databases (e.g., JSTOR Global Plants), university library digitization programs, monographs and floras (Frodin, 2001), and public websites (e.g., Wikispecies). We argue that, when combined with targeted field campaigns in undersampled regions, the mobilization of already available but unstructured information in texts from the above resources may help to fill gaps in pertinent trait databases and hence boost the availability of trait data for macroecological analyses.

## AUTHOR CONTRIBUTIONS

V.D., H.K., and P.W. conceived the ideas of the paper. V.D. and P.W. designed the methodology with helpful discussions with R.K., H.K., Ph.W., and A.Z. V.D. performed the analyses and created the visualizations. V.D. led the writing of the first draft of the manuscript. All authors contributed critically to the drafts and gave final approval for publication.

## Supporting information


**Appendix S1.** Pipeline example.
**Appendix S2.** Comparison of the Plants of the World Online and Wikipedia datasets.
**Appendix S3.** Regex keywords.
**Appendix S4.** Model implementation.
**Appendix S5.** Comparison of explicit and implicit wording in descriptions.
**Appendix S6.** Inter‐dataset evaluation.
**Appendix S7.** Analysis of probabilistic predictions.
**Appendix S8.** Analysis of numerical trait predictions.

**Appendix S9.** Predicted trait values for the Plants of the World Online dataset.

**Appendix S10.** Predicted trait values for the Wikipedia dataset.

## Data Availability

The textual species descriptions from Plants of the World Online and Wikipedia, and the trait data from the Global Inventory of Floras and Traits, are available for download using the taxize and GIFT R packages (Chamberlain et al., 2020; Denelle et al., 2023). The predicted trait values, with their associated confidence values using the DistilBERT model on both datasets, are available in the Supporting Information (Appendices S9 and S10). However, it should be acknowledged that further research is needed before these methods and their predictions can be considered reliable in practice. Rigorous safety evaluations are essential to prevent the introduction and reinforcement of biases in downstream tasks and ecological analyses, which could potentially result in more unintended consequences than benefits. Users should exercise caution when using these predicted trait values, as they are not yet validated for research purposes and should not be treated as definitive trait data. Annotated Python code for preprocessing of the data; training and evaluation of the keyword search, logistic regression, and large language models; and trait extraction with the trained models is available on GitHub (https://github.com/ViktorDomazetoski/NLP-Plant-Traits). The fine‐tuned large language models are available on HuggingFace (https://huggingface.co/ViktorDo).
