# Supplementary material for: Using large language models to extract plant functional traits from unstructured text
Source: Appl Plant Sci. 2025 Jun 3;13(3):e70011. doi: 10.1002/aps3.70011 (PMC12188621; doi:10.1002/aps3.70011)
Supplement: Supplementary file 1 — Appendix S1. Pipeline example. Appendix S2. Comparison of the Plants of the World Online and Wikipedia datasets. Appendix S3. Regex keywords. Appendix S4. Model implementation. Appendix S5. Comparison of explicit and implicit wording in descriptions. Appendix S6. Inter‐dataset evaluation. Appendix S7. Analysis of probabilistic predictions. Appendix S8. Analysis of numerical trait predictions. [file APS3-13-e70011-s002.docx]

# Supporting Information for “Using large language models to extract plant functional traits from unstructured text”

Authors: Viktor Domazetoski, Holger Kreft, Helena Bestova, Philipp Wieder, Radoslav Koynov, Alireza Zarei, Patrick Weigelt

##
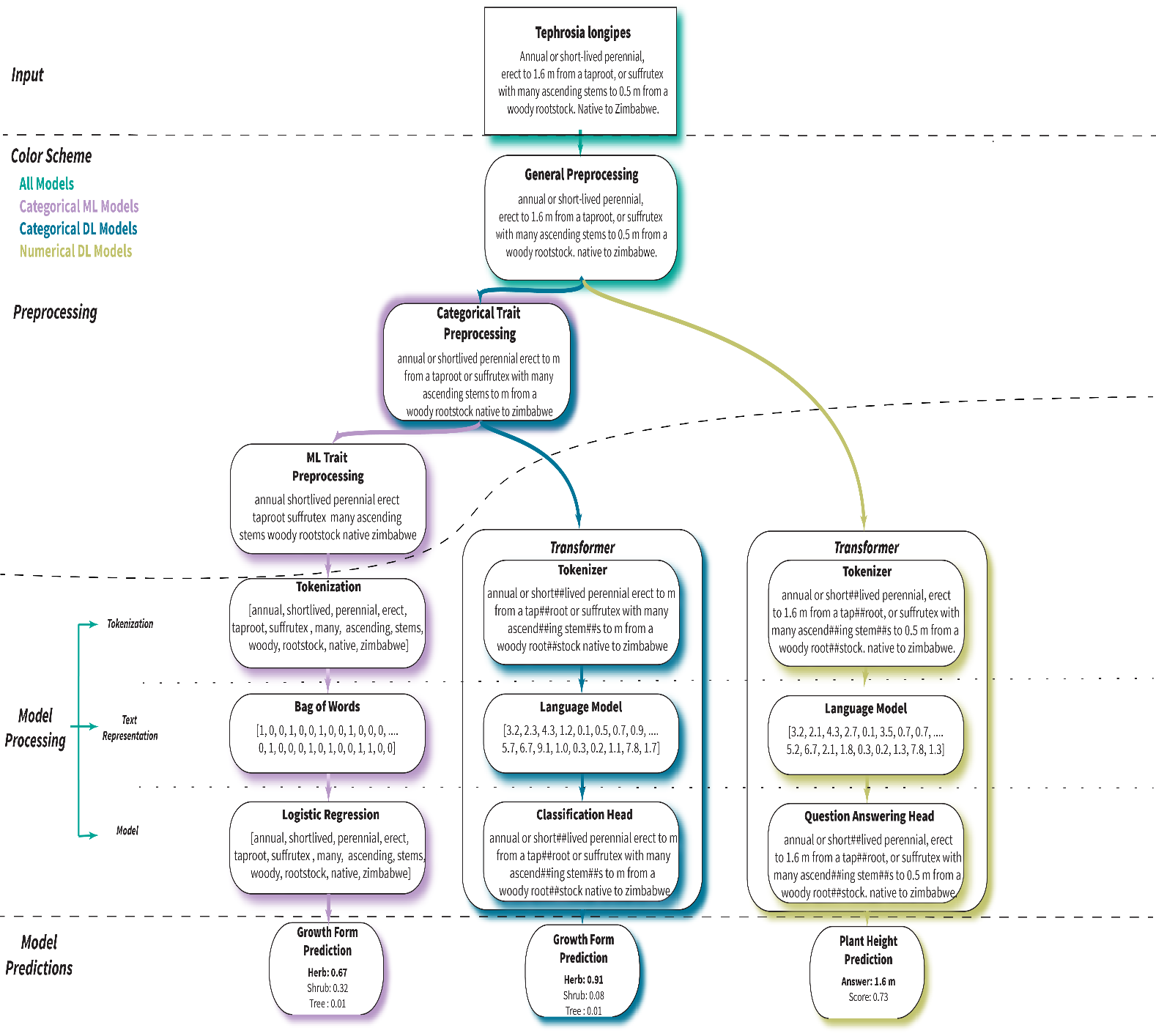
Appendix S1. Pipeline example.

**Figure S1.** Example application of the proposed NLP pipeline used for the prediction of categorical and numerical traits for one species. The description first goes through a general preprocessing pipeline where it is lowercased and all abnormalities are removed. Then for the categorical traits, the digits and punctuation are further removed and only for the categorical ML pipeline, stop words are also removed. The preprocessed description is then tokenized based on the requirements of the model and is embedded in a vector space using the bag of words model or a large language model. Using this embedding as input for a classification or question answering model, the model associates weights to each trait value (only one shown here). Using these weights, the final prediction is made and an associated confidence score is provided.

## Appendix S2. Comparison of the Plants of the World Online and Wikipedia datasets.

## Plants of the World Online Dataset

Plants of the World Online (<http://www.plantsoftheworldonline.org/>) is an online platform established by Kew Royal Botanic Gardens, dedicated to digitizing and sharing comprehensive data on the world’s flora. The Plants of the World Online scrape comprised 288,254 descriptions encompassing 59,151 species, categorized into 251 distinct description types, originating from 15,023 different sources. On average, each species is associated with approximately 4.86 descriptions (standard deviation 5.84), though due to an uneven distribution, the median count is 2 descriptions per species. To create the POWO dataset, these descriptions were organized on a per-species-per-source basis. On average, the descriptions in this aggregated POWO dataset consist of 118 words, however, the median is only 6 words (Figure S2a). This discrepancy is primarily attributed to the fact that a significant portion (46.9%) of the descriptions contain fewer than five words, necessitating manual examination to determine if they contain the trait data required for training and evaluating models. The entire corpus of descriptions comprises 52,336 tokens, with 44% occurring only once and 80% occurring fewer than 10 times (Figure S2b) The geographic species distribution of the dataset closely mirrors the actual distribution of plant species (Figure S3a). However, the average geographic word count reveals variation, with the Amazon region, which has the highest number of species descriptions, featuring the chfewest words, averaging under 50 words per species (Figure S4a). In contrast, descriptions in Africa are much more extensive, as the initial purpose of Plants of the World Online was to document these species. In terms of trait coverage, the growth form, epiphyte, and climber traits have a substantial representation, surpassing 70%, while numerical traits, such as leaf length and width, have a much more limited coverage, with only a few thousand descriptions available (Table S1).

**Table S1.** Trait and trait value coverage for the POWO dataset.

## Wikipedia Dataset

The English-language Wikipedia corpus scrape comprises 194,994 descriptions related to 55,631 species, organized into 7,903 description types, sourced from 22,035 unique authors. On average, there are about 3.5 descriptions for each species, with a standard deviation of 3.1. However, due to a skewed distribution, the median number of descriptions per species stands at 3. These descriptions were consolidated into a single description per species per source, resulting in the formation of the WIKI dataset, encompassing 55,631 unique entries. The descriptions of the WIKI dataset include an average of 198 words, with a median length of 98 words (Figure S2a). In total, the corpus of descriptions consists of 211,645 tokens, with 48% of them occurring only once and 84% occurring less than 10 times (Figure S2b). Many of these tokens with few occurrences contain taxonomic information about the species, such as family, genus, or binomial names. The geographic distribution of species in the dataset exhibits a bias towards English-speaking countries, aligning with the expectations from the English Wikipedia corpus (Figure S3b). The average word count displays a more even distribution globally (Figure S4b). Nevertheless, some regions, such as the Amazon, remain relatively unrepresented in the dataset.

This observation suggests that sourcing data from Wikipedia in various languages could potentially augment the dataset with new species and information. Trait coverage distribution in the WIKI dataset is similar to the POWO dataset, with generally higher coverage, as the data scraping specifically targeted species with corresponding label data in GIFT (Table S2).

**Table S2.** Trait and trait value coverage for the WIKI dataset.

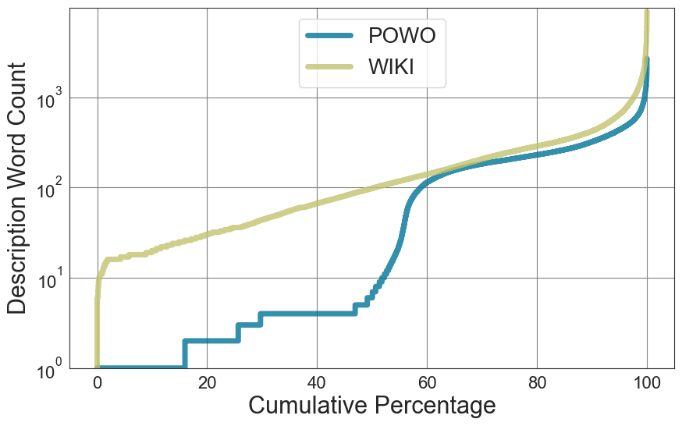


a)


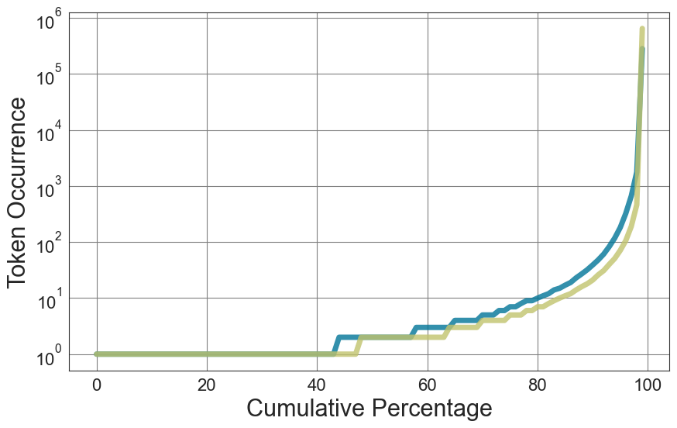


b)

**Figure S2.** Cumulative percentage of the log-description word count (a) and log-token occurrence (b) of the Plants of the World Online (POWO) (blue) and Wikipedia (WIKI) (olive) datasets.


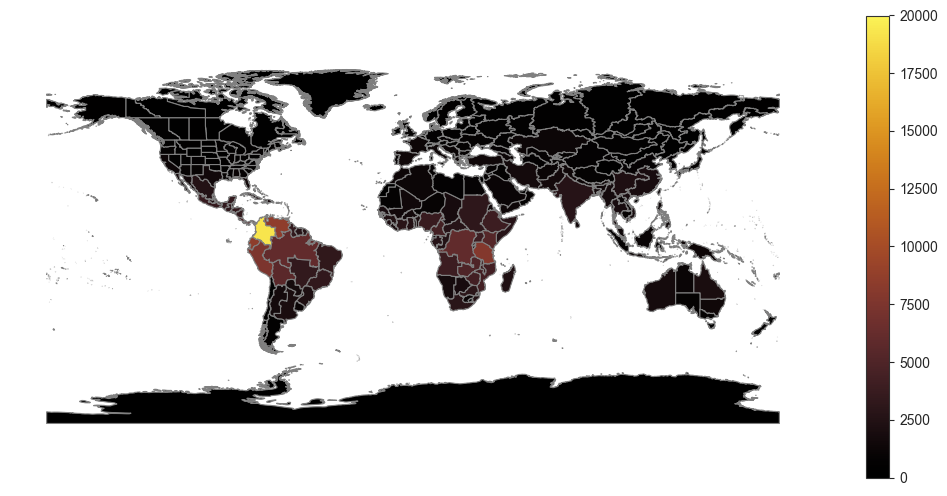

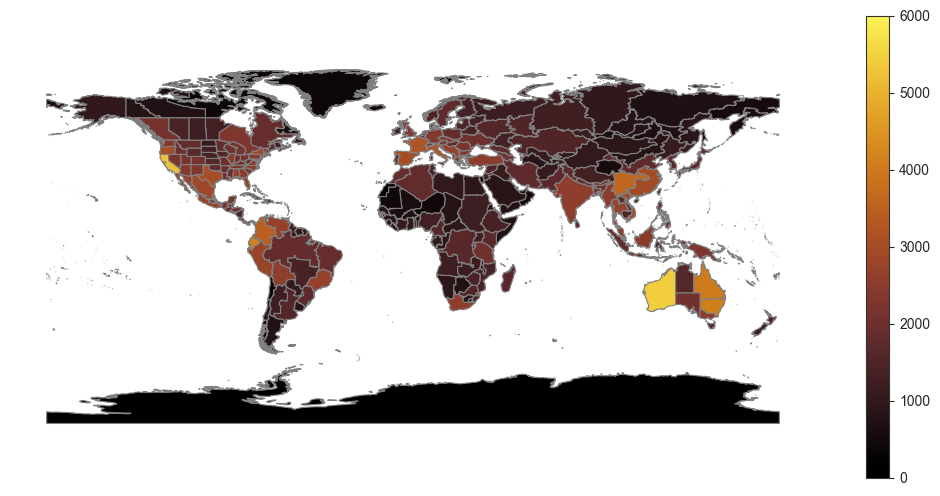


b)

a)

**Figure S3.** Geographic distribution of the species in the Plants of the World Online (POWO) (a) and Wikipedia (WIKI) (b) datasets. To map the species distribution, we combined the 59,151 species of the POWO and 55,631 species of the WIKI dataset with their level 3 botanical region of the World Checklist of Vascular Plants (Govaerts et al., 2021) and then calculated the species richness for each botanical region.

###
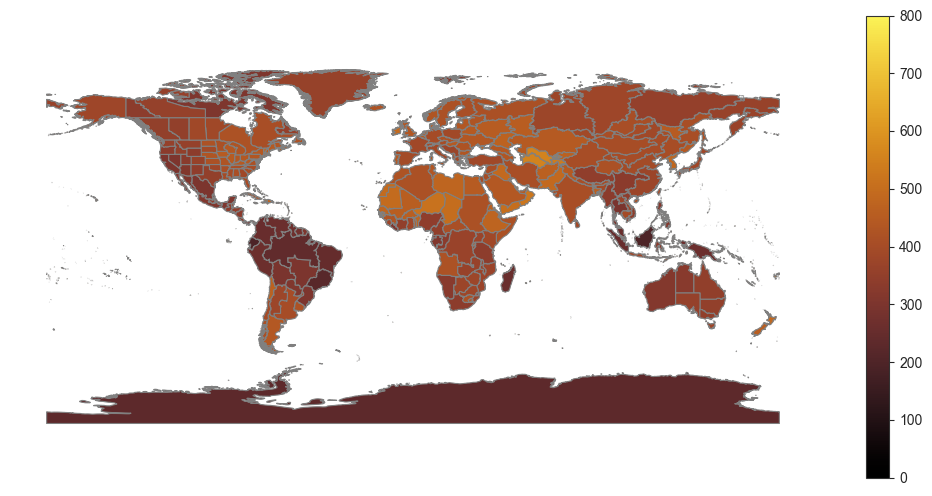

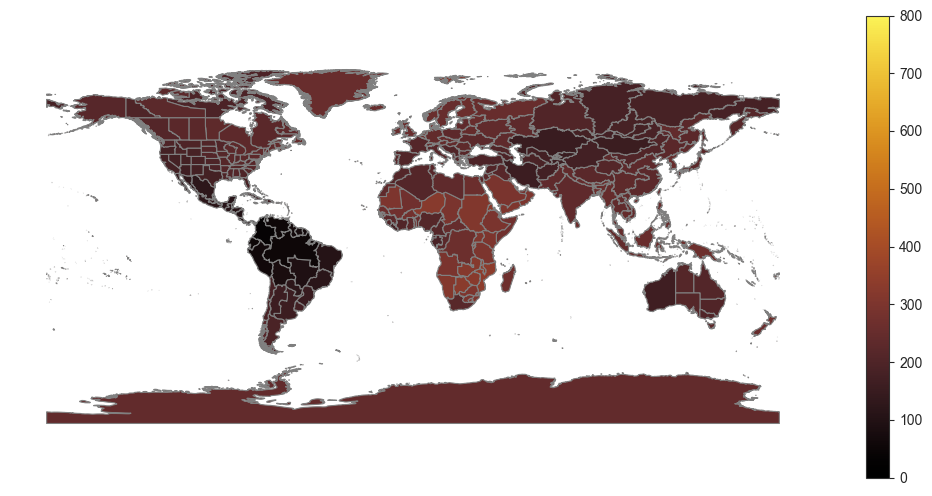


b)

a)

**Figure S4.** Average description word count per botanical region in the Plants of the World Online (POWO) (a) and Wikipedia (WIKI) (b) datasets. To map the average word count, we combined the 59,151 species of the POWO and 55,631 species of the WIKI dataset with their level 3 botanical region of the World Checklist of Vascular Plants (Govaerts et al., 2021) and then calculated the average word count for each botanical region.

Govaerts, R., Nic Lughadha, E., Black, N., Turner, R., & Paton, A. (2021). The World Checklist of Vascular Plants, a continuously updated resource for exploring global plant diversity. Scientific Data, 8(1), 215.

### Appendix S3. Regex keywords.

**Table S3.** Trait values and their associated dictionary used in the keyword search model.

| **Growth Form** | Herb | herb, herbaceous, forb, orchid, graminoid, herbaceous perennial, reed, rush, grass, grasslike, sedge, geophyte, annual, biennial, hemicryptophyte, cryptophyte, therophyte |
| --- | --- | --- |
|  | Shrub | shrub, subshrub, undershrub, bush, shrublet, cactus, suffrutex, shrubby, chamaephyte |
|  | Tree | tree, mallet, arbor, treelet |
| **Epiphyte** | Epiphytic | epiphyte, epiphytic, holoepiphyte, hemiepiphyte |
|  | Terrestrial | terrestrial, tree |
| **Climber** | Climber | climber, climbing, vine, liana, liane, woodytwiner, shrubbytwiner, woodyclimber, twiner |
|  | Self-supporting | self-supporting, supporting, tree |
| **Life cycle** | Annual | annual, winter-annual, summer-annual, ephemeral, short-lived |
|  | Perennial | perennial, long-lived, evergreen, tree |
| **Life Form** | Phanerophyte | phanerophyte, nanophanerophyte, tree |
|  | Chamaephyte | chamaephyte |
|  | Hemicryptophyte | hemicryptophyte |
|  | Cryptophyte | cryptophyte, hydrophyte, geophyte, helophyte |
|  | Therophyte | therophyte, annual, biennial, winter-annual, summer-annual, ephemeral, short-lived |

## Appendix S4. Model implementation.

The codebase was written in Python v. 3.9.13 and either directly or indirectly relied heavily on the NumPy (Harris et al., 2020) and pandas (McKinney, 2011) libraries for the organization and processing of data. Text analysis and preprocessing were done using the NLTK python library (Loper & Bird, 2002). We implemented the bag-of-words (BOW) and logistic regression model using the scikit-learn ML library v. 1.1.3 (Pedregosa et al., 2011). We trained and evaluated the large language models using the Huggingface’s transformers library v. 4.28.0 (Wolf et al., 2019). The models were trained using Google Colab and Kaggle on the freely available T4 GPUs. The models were trained for 3 epochs with a batch size of 16 and a maximum sequence length of 512 tokens. The default learning rate was set to 2e^-5^ and a weight decay of 0.01 was applied. The visualizations were done using the Python Matplotlib (Hunter, 2007) and Seaborn libraries (Waskom, 2021). The entire codebase for the manuscript is open-source and available on GitHub (<https://github.com/ViktorDomazetoski/NLP-Plant-Traits>).

**REFERENCES**

Harris, C. R., Millman, K. J., Van Der Walt, S. J., Gommers, R., Virtanen, P., Cournapeau, D., ... & Oliphant, T. E. (2020). Array programming with NumPy. Nature, 585(7825), 357-362.

Hunter, J. D. (2007). Matplotlib: A 2D graphics environment. Computing in science & engineering, 9(03), 90-95.

Loper, E., & Bird, S. (2002). NLTK: The natural language toolkit. arXiv Cs/0205028 [Preprint]. Available at <https://doi.org/10.48550/arXiv.cs/0205028> [posted 17 May 2022; accessed 2 May 2025].

McKinney, W. (2011). pandas: a foundational Python library for data analysis and statistics. Python for high performance and scientific computing, 14(9), 1-9.

Pedregosa, F., Varoquaux, G., Gramfort, A., Michel, V., Thirion, B., Grisel, O., ... & Duchesnay, É. (2011). Scikit-learn: Machine learning in Python. Journal of machine Learning research, 12, 2825-2830.

Waskom, M. L. (2021). Seaborn: statistical data visualization. Journal of Open Source Software, 6(60), 3021.

Wolf, T., Debut, L., Sanh, V., Chaumond, J., Delangue, C., Moi, A., ... & Rush, A. M. (2019). Huggingface's transformers: State-of-the-art natural language processing. ArXiv 1910.03771 [Preprint]. Available at <https://doi.org/10.48550/arXiv.1910.03771> [posted 9 October 2019; accessed 2 May 2025].

## Appendix S5. Comparison of explicit and implicit wording in descriptions.

**Table S4.** Species’ descriptions and the associated label for the growth form trait in the GIFT database, the keyword search model prediction, and the DistilBERT model predictions above 50% confidence. Correct predictions are labeled in green and incorrect predictions are labeled in red. It should be noted that the trait “correctness” is only based on the label of the species in GIFT, not the label as it would be extracted from the description.

| **Species Name** | **Description** | **Growth Form Label in GIFT** | **Keyword Predictions** | **DistilBERT Predictions (Confidence %)** |
| --- | --- | --- | --- | --- |
| Amelanchier pallida | Amelanchier pallida, the pale serviceberry or western serviceberry, is a species of Amelanchier native to the US states of California and Arizona. They are shrubs or small trees reaching 10 ft (3 m), with attractive blue-green foliage. They typically grow in mountains up to 11,000 ft (3,400 m) above sea level, generally alongside streams. Native Americans used to dry the berries for winter provisions, and they can be made into a jam. | Shrub | Shrub  Tree | Tree (98.1%) |
| Asimina incana | Asimina incana is a species of pawpaw (genus Asimina, family Annonaceae). It is a shrub that grows to a height of 1.5 metres (4 ft 11 in). Its leaves are 3-6 millimetres (0.12–0.24 in) long and leathery. It usually grows 1–4 flowers per node. Its pollen is shed as permanent tetrads. | Shrub | Shrub | Tree (97.4%) |
| Amphitecna macrophylla | Amphitecna macrophylla, commonly known as black calabash or chaff-bush, is a species of plant in the family Bignoniaceae. It is found in small patches of Mexico and Guatemala. It can reach a height of 15 to 30 feet (4.6–9.1 m). It is drought tolerant and is hardy to USDA Hardiness Zone 10b. | Tree | Shrub | Shrub (87.8%)  Tree (51.6%) |
| Yucca intermedia | Yucca intermedia McKelvey is a species in the family Asparagaceae, with the common name intermediate Yucca. It is a relatively small plant forming clumps of rosettes. It is native to juniper-pinyon woodlands of the US State of New Mexico, at an elevation of 1,000–2,100 m (3,300–6,900 ft). | Herb |  | Herb (56.1%)  Shrub (56.2%) |
| Allium pevtzovii | Allium pevtzovii is a Chinese species of wild onion found only in the southwestern part of Xinjiang Uygur Province in extreme western China.Allium pevtzovii has a cluster of narrow, cylindrical bulbs. Scapes are up to 25 cm (9.8 in) tall. Umbel is a dense cluster of red, lustrous flowers. | Herb |  | Herb (99.5%) |
| Acacia castanostegia | Acacia castanostegia is a shrub belonging to the genus Acacia and the subgenus Phyllodineae. It is native to an area in the Wheatbelt and the Goldfields-Esperance regions of Western Australia. The dense, rounded and prickly shrub typically grows to a height of 0.2 to 0.5 metres (1–2 ft). | Shrub | Shrub | Shrub (97.6%) |
| Aneilema beniniense | A robust straggling herb up to 2–3 ft. high, producing flbrous roots from the lower nodes; Leaves mostly elliptic to ovate, rarely all narrow and lanceolate; Petals white to lilac; Flowers open from 7 a.m. to noon; Inflorescence dense, about 1–2 1/2 in. across in fruit; Usually subglabrous, rarely rather densely pubescent | Herb | Herb | Herb  (96%) |
| Thunbergia cynanchifolia | Pure white flowers 3/4 in. long, the stems either glabrous or spreading-pilose. A slender climbing plant, on trees, etc. | Herb | Tree | Herb (66.7%) |

To analyze the impact of explicit vs implicit trait mentions on the performance of the LLM models, we calculated the macro precision and recall for the keyword search and DistilBERT model on a subset of the test dataset for which the keyword search was able to find a match vs a subset for which it was not able to find a match, irrespective of whether that match was correct or not. First, we can see that the performance of the keyword search is not as high as we would expect in the matched subset, mostly due to the data fusion issue talked about in the discussion. Second, we can see that DistilBERT sees an average decrease of 6.03% in precision and 18.21% in recall on the POWO, and 4% in precision and 8.65% in recall on the WIKI dataset. This might be seen both as good and as bad: On one side it means that the models can use implicit information and natural language to make predictions, as indicated by a lower decrease of performance in the WIKI dataset. However, it might also mean that the model has learned accidental associations based on the training data, where words that do not necessarily carry trait information, but have some relation to the traits (such as countries or habitats) have been used to make the prediction.


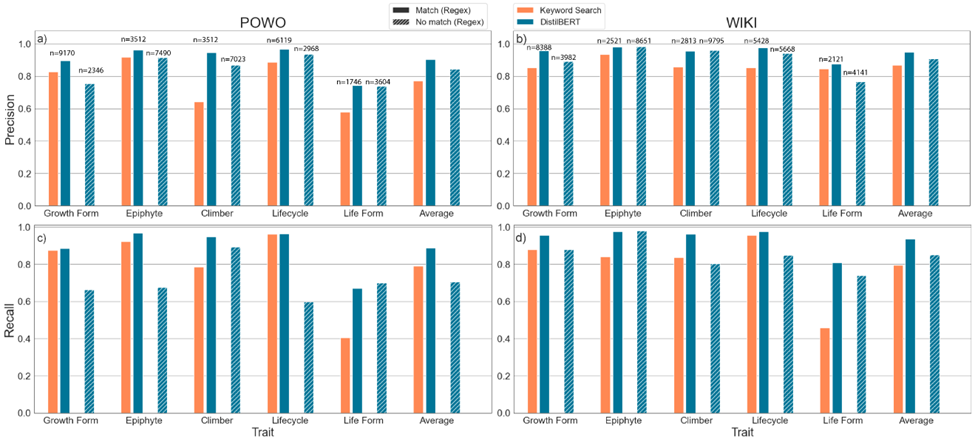


**Figure S5.** Model comparison across the Plants of the World Online (a, c) and Wikipedia dataset (b, d) when the keyword search was able to find a match (solid) vs. when it was not able to find a match (dashed). Precision (a, b) and recall (c, d) for the categorical traits are shown for the keyword search (orange) and DistilBERT (blue). The number of descriptions for each subset are shown above the bars for each trait and dataset.

## Appendix S6. Inter-dataset evaluation.

In order to effectively utilize these models in practical applications, it is crucial to assess their performance on datasets with distinct characteristics from those used for training. To address this, we conducted an inter-dataset evaluation, where models trained on the training set of one dataset were evaluated on the test set of the remaining two datasets (Figure S6). Along the diagonal lie the intra-dataset scores. The results revealed that the models trained on POWO and POWO_MGH demonstrated nearly identical intra-dataset performance. This implies that, particularly for categorical models, utilizing only the POWO categories directly relevant to a specific trait does not significantly enhance model performance. However, in the case of inter-dataset scores, the variation in performance between datasets remained relatively consistent across traits, with one notable exception: the life form trait. The observed disparity can be attributed to the limited data available in the POWO_MGH dataset for this specific trait. When evaluating inter-dataset scores between POWO and WIKI, a more pronounced decline in performance was evident across all traits. This decrease in performance averaged 8.9% in precision and 23% in recall when POWO served as the training dataset, and 13.8% in precision and 8.5% in recall when WIKI was the training dataset. The most substantial decrease was again observed in the life form trait, with precision decreasing by 25% (14%) and recall by 26% (13%) for the POWO (WIKI) datasets. These findings underscore the importance of exercising caution when drawing inferences from new data, as the model's performance is intricately tied to the characteristics of the training dataset.


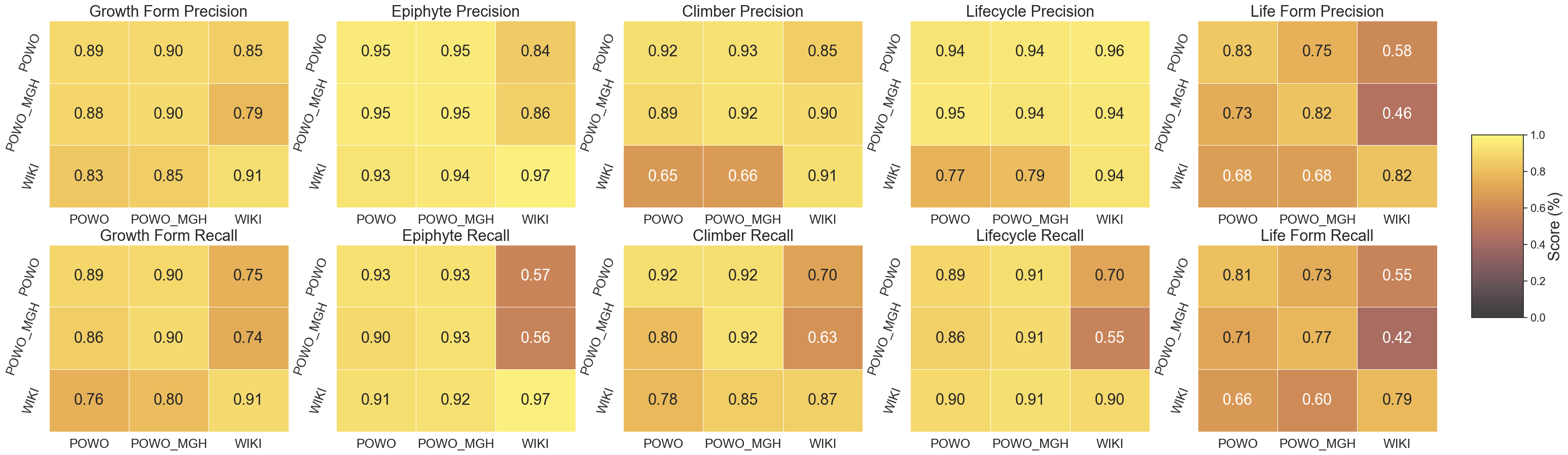


**Figure S6.** Inter-dataset precision (first row) and recall (second row) scores using the DistilBERT model for the categorical traits of interest (columns). Within each matrix, each row of the heatmap represents the dataset used in training, while each column represents the dataset used for testing. The values on the diagonal correspond to the values of the intra-dataset models discussed in the main text (under Results, Categorical Traits).

## Appendix S7. Analysis of probabilistic predictions.

We also explored the impact of the probability threshold (*t*) on model performance. This threshold serves as a versatile tool, enabling users to tailor results according to their specific requirements. Raising the threshold allows users to achieve a higher precision at the expense of a reduced recall, while lowering the threshold yields the opposite effect. To gain insights into this behavior, we constructed precision–recall curves. These curves provide a visual representation of how the model's performance evolves and how the trade-off between precision and recall varies at different threshold settings (see Figure S7).


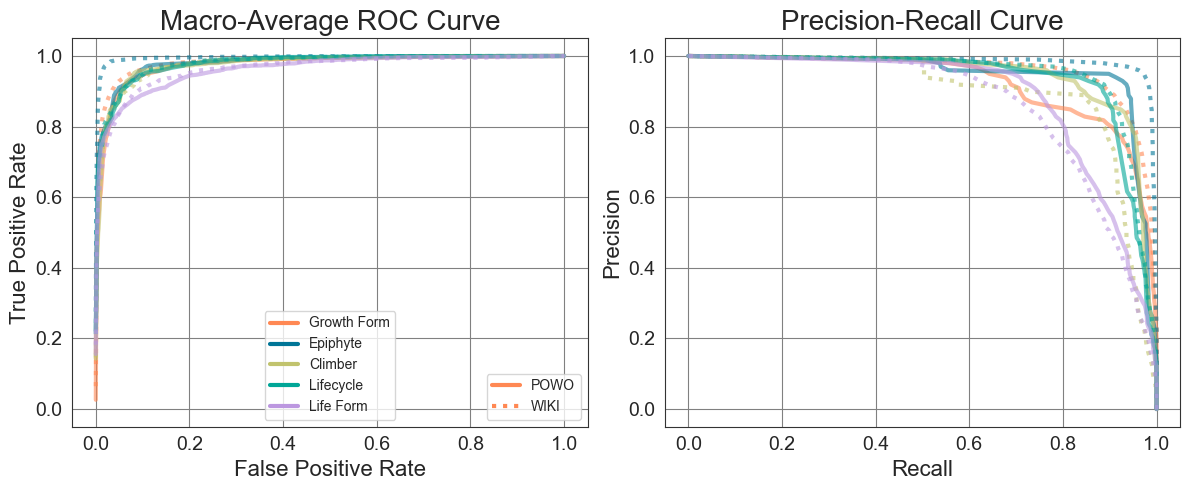


b)

a)

**Figure S7.** Macro-average ROC curve (a) and Precision–Recall curve (b) for the DistilBERT model on the Plants of the World Online (POWO) (solid line) and Wikipedia (WIKI) (dotted line) datasets.

b)

a)

For a more comprehensive understanding of how specific threshold values affect the models, we generated precision and recall curves across a range of threshold values from 0 and 1 (Figure S8). As anticipated, precision displayed a positive correlation with the threshold, peaking at optimal values around *t* = 0.8. However, for select traits (epiphyte, climber, life cycle), the precision curve plateaued at around *t* = 0.4, while for the growth form and life form traits, precision continued to rise until the higher end of the threshold range. Conversely, recall exhibited the opposite pattern, with a more consistent performance across the five traits of interest.


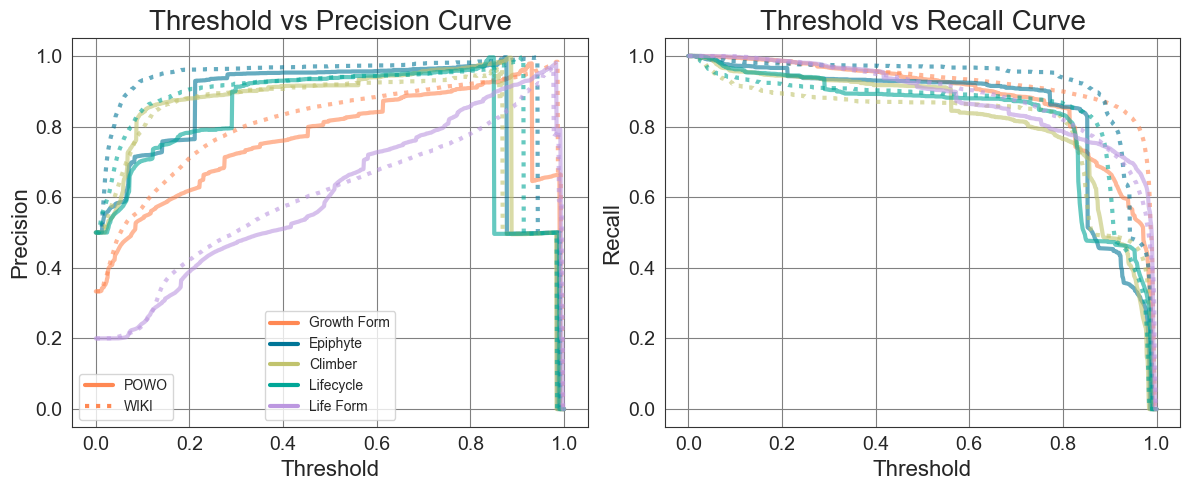


b)

a)

**Figure S8.** Threshold vs Precision (a) and Recall (b) for the DistilBERT model on the Plants of the World Online (POWO) (solid line) and Wikipedia (WIKI) (dotted line) datasets.

Finally, we contrast the results reported in our paper, which were obtained by taking the trait prediction with the highest probability (argMax), with the model’s performance when applying probabilistic thresholds of *t =* 0.5 and *t =* 0.8 (Figure S9). We observe that when using *t =* 0.5, the results generally resemble those of the argMax approach, albeit sometimes at the expense of precision in favor of achieving higher recall, such as in the growth form and life form traits. On the other hand, *t* = 0.8 yields a substantial increase in precision, at the cost of a reduced recall, making it valuable for scenarios where the data quality must be stringent and manual verifications are limited.


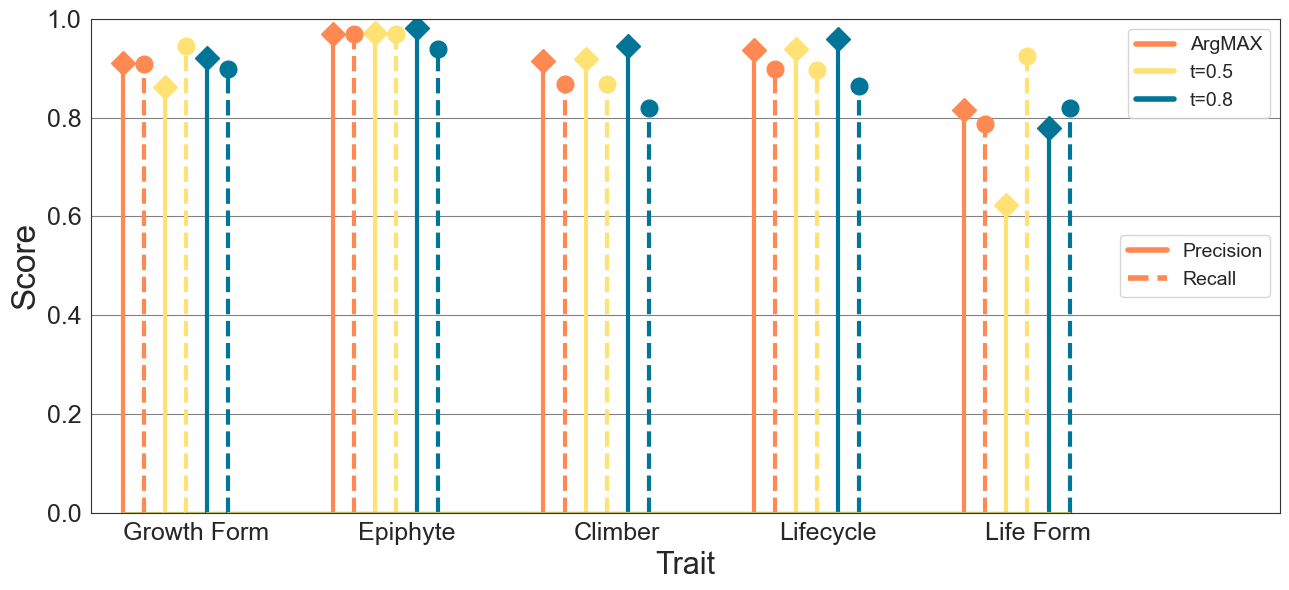


b)

a)

**
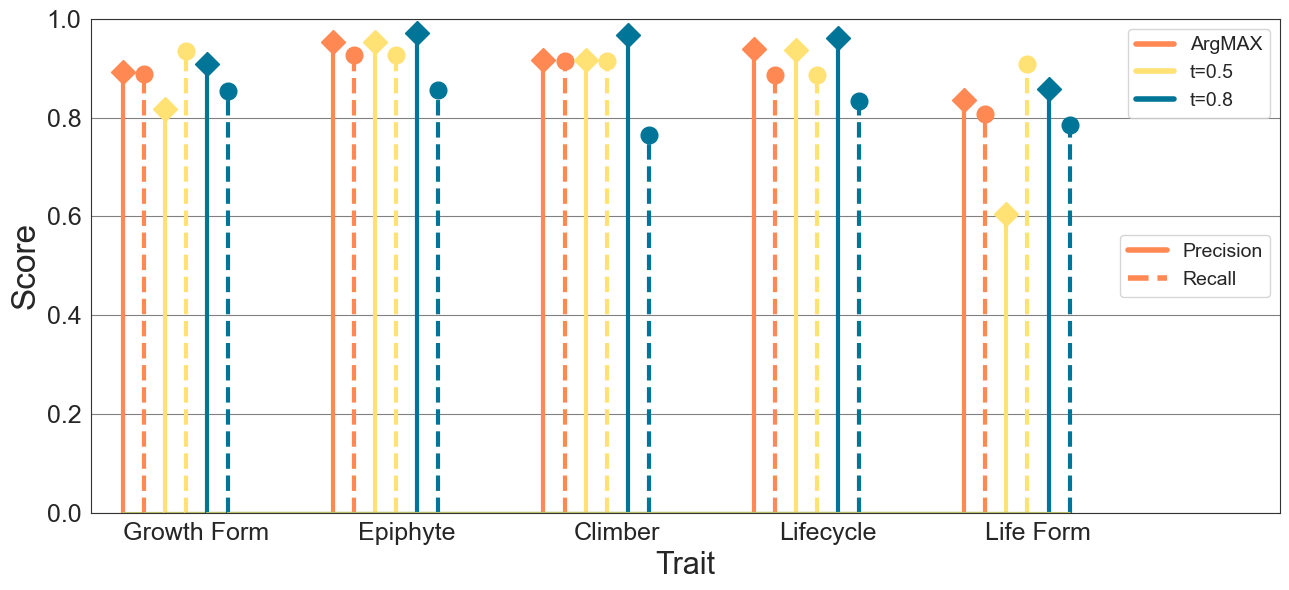
Figure S9.** Precision (solid line) and Recall (dashed line) for the DistilBERT model on the Plants of the World Online (POWO) (a) and Wikipedia (WIKI) (b) datasets when using an argMax approach (orange), a threshold *t* = 0.5 (yellow), and *t* = 0.8 (blue).

b)

## Appendix S8. Analysis of numerical trait predictions.


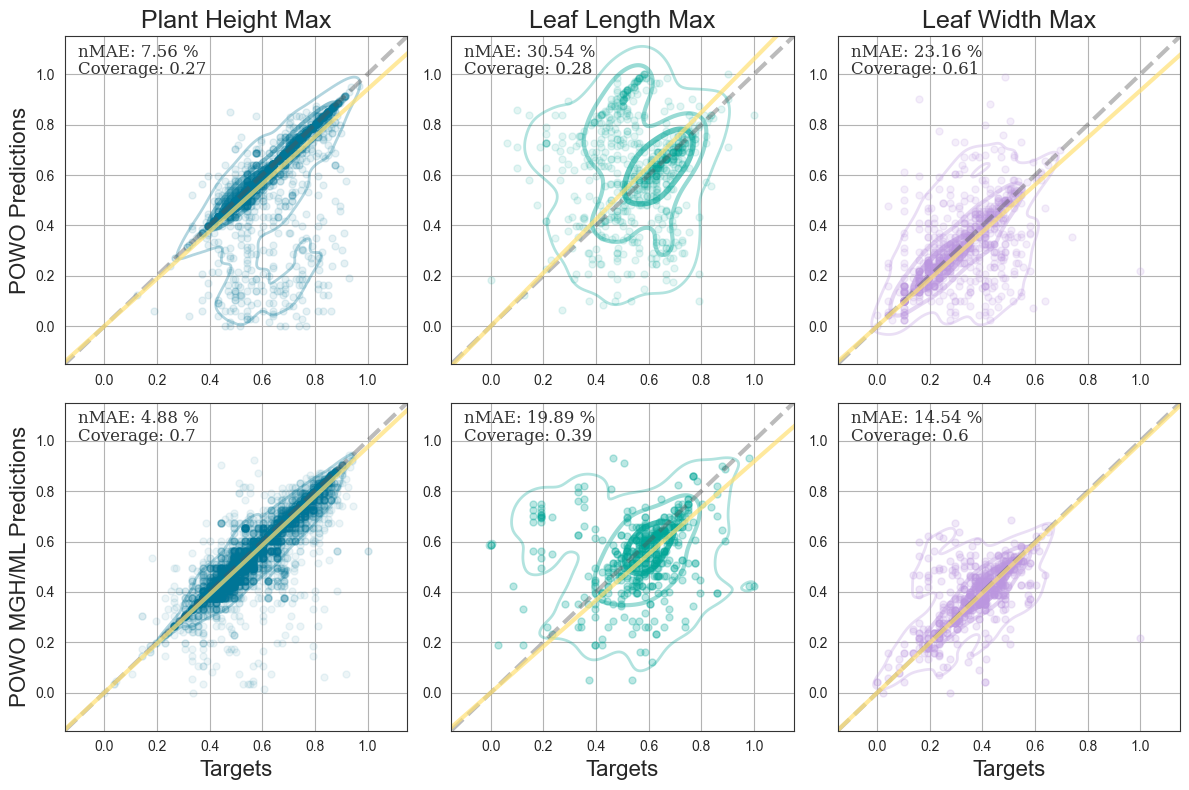


**Figure S10.** Observed vs. predicted numerical traits for the DistilBERT model on the aggregated Plants of the World Online and trait-specific Plants of the World Online (descriptions utilizing only specific Plants of the World Online categories: MGH: Morphology_General_Habit; ML: Morphology_Leaf) datasets. The numerical traits are represented as plant height (blue), leaf length (cyan), and leaf width (violet). The 95% and 50% kernel density estimates are also shown as polygons in the corresponding trait color. The 1:1 line (gray dashed) and the regression line between the observed and predicted values (yellow solid) are also shown.
